# Pharmaceutical Activities, Services, Medicines, and Products at Community Pharmacies in the Qassim Region of Saudi Arabia: Findings and Implications for the Future

**DOI:** 10.7759/cureus.67957

**Published:** 2024-08-27

**Authors:** Alian A Alrasheedy

**Affiliations:** 1 Department of Pharmacy Practice, College of Pharmacy, Qassim University, Qassim, SAU

**Keywords:** saudi arabia, scope of practice, public health, pharmaceutical activities, health policy, community pharmacists

## Abstract

Introduction

Community pharmacies are highly accessible and provide access to several services to patients and community members. Recently, several developments have been made in Saudi Arabia to enhance the roles and contributions of community pharmacists to the healthcare system, including expanding their scope of practice. This study aims to evaluate the current pharmaceutical activities, medicines, products, and services offered by community pharmacies.

Methods

This was a cross-sectional questionnaire-based descriptive study. The questionnaire consisted of three sections. The first section comprised the demographic characteristics of the participants. The second section explored the types of pharmaceuticals and products sold by community pharmacies, while the third section explored the current professional services and activities offered by pharmacies. This study was conducted among community pharmacists in the Qassim region of Saudi Arabia.

Results

Overall, 109/115 community pharmacies participated in the study, yielding a response rate of 94.78%. Most participants (97.25%) were men, and 42.20% were 31-40 years old. All pharmacies (100%) dispensed prescriptions and provided over-the-counter medicines, self-care therapeutics, vitamins, minerals, and dietary supplements. However, only a few pharmacies had controlled and narcotic medicine services (5.50%) and supplied vaccines (3.67%). Almost all pharmacies provided access to herbal products (97.25%), self-diagnostic test/home-test kits (97.25%), first-aid kits (95.41%), and medical equipment and devices and their accessories (89.91%). All pharmacies (100%) sold health-related products, including oral, skin, and hair care products. All pharmacists (100%) provided medication counseling. However, e-prescription services (Wasfaty) were provided in only 55.96% of the pharmacies. Most pharmacists provided health education and promotion (95.41%), management of minor ailments (88.99%), and patient training on the use of medical devices (92.66%). Other pharmaceutical activities included travel health advice (52.29%) and smoking cessation (31.19%). However, patient care services, including vaccination services (0.92%), patient assessment and monitoring services (0.92%), and point-of-care (POC) testing (0.92%), were limited. Additional services included online shopping (66.97%), home delivery of medicines (54.13%), and instant/live chat communication with pharmacies (70.64%).

Conclusion

Community pharmacies play a crucial role in Saudi Arabia’s healthcare system. These include providing access to medicines, medical equipment, and various products related to health and wellness. Community pharmacies provide public health services. However, their clinical services are limited. Consequently, a holistic strategy involving all stakeholders is required to further enhance the role of community pharmacists and better utilize their skills and training to provide preventive healthcare services and optimize medication therapy in primary care settings.

## Introduction

Background

Community pharmacies are easily accessible to patients and highly visible to the public [1-3]. This is because of their extended opening hours and locations in communities, and no appointment is needed for consultations, advice, or access to medicines [4-6]. Consequently, community pharmacists play a vital role in contemporary healthcare systems by providing access to medicines, pharmaceuticals, and healthcare services to patients and the community. Moreover, during the COVID-19 pandemic, community pharmacists played a significant role in combating the pandemic and supporting the healthcare system by providing and maintaining essential healthcare services to communities and patients, including a stable supply of medicines. In this critical period, most pharmacies remain open to delivering services or extending their working hours to meet the increase in workload in response to patient needs [7-9]. The pandemic has demonstrated several roles that pharmacists could play in health emergencies, public health crises, and critical situations. These include COVID-19 testing services, COVID-19 vaccine administration, supply of personal protective equipment, including face masks, gloves, and other items such as hand sanitizers and disinfectant products, patient education and advice, and telehealth services [1,7,8,10]. 

The traditional role of community pharmacies includes dispensing and supplying prescription and over-the-counter (OTC) medicines. However, other professional activities, services, and regulated scopes of practice vary widely across countries and healthcare systems [11-15]. Recent developments have created new opportunities for community pharmacists to provide patient care services and expand their roles, especially in high-income countries. These include immunization services, chronic disease management (e.g., diabetes, hypertension, asthma, and obesity), reproductive health services, oral health consultations, medication review services, and support services for drug addiction [2,16-19].

In Saudi Arabia, community pharmacy practices typically focus on traditional roles, including dispensing prescription medicines and OTC medicines and providing brief standard counseling with limited patient care services [20,21]. Recently, initiatives and developments have been introduced to enhance the role of community pharmacists and their contributions to the healthcare system. These include the national electronic prescribing service (Wasfaty), in which community pharmacies play key roles in providing pharmaceutical services and dispensing medications for all patients with e-prescriptions from government primary healthcare centers (PHC) and hospitals (i.e., dispensing medicines and pharmaceutical services were transferred to community pharmacies) [22]. Other developments include new regulations to expand the scope of practice [23] and graduates with a Doctor of Pharmacy (PharmD) degree joining this sector [24]. However, there is a paucity of studies that evaluated the current scope of practice, professional services, pharmaceutical activities, trends in regulations and policies, or their impact on community pharmacy practices. Consequently, this study aimed to fill this gap in the literature, provide a comprehensive overview of the professional scope of practice and evaluate current professional activities, medicines, products, and services provided by community pharmacies. The current study provides health decision-makers with valuable data regarding the community pharmacy sector. Consequently, the study findings can be utilized to guide the planning and improvement of pharmaceutical services in community pharmacies in Saudi Arabia. This article is organized into two parts. The first part provides a comprehensive overview of the scope of the practice (professional scope and regulated activities), and the second part presents a cross-sectional study evaluating the current pharmaceutical activities, medicines, products, and services provided by community pharmacies (i.e., the extent of implementation of the services, including types and availability).

Overview of the professional scope of practice (regulated activities)

There have been major developments in laws and regulations since the 1970s to meet the healthcare system requirements and the progress of the pharmacy profession [25-34]. The first law related to the practices of the pharmacy profession in Saudi Arabia dates back to 1978. This is called the Law of Practice of Pharmacy Profession, and Trading of medicines and medical products [25]. It aimed to regulate the practices of pharmacies and the trading of medicines and other medical products in the pharmaceutical market. In 2004, this law was replaced with the Law of Pharmaceutical Institutions and Pharmaceutical Products [26]. The executive regulations of this law were introduced in 2005 and updated in 2019 [28, 29]. In the executive regulations issued in 2019, the scope of community pharmacists was expanded, and they were legally authorized to provide a set of patient care services beyond traditional dispensing services. The 2004 law and its executive regulations (2019) were replaced in 2020 with the laws of pharmaceutical institutions, pharmaceutical and herbal products, and executive regulations (2020) [27]. Another key law related to the practice of community pharmacies is the Law of Health Professions in Saudi Arabia, issued in 2005, and its executive regulations issued in 2006 [30,31]. The Executive Regulations of the Practice of Health Professions were updated twice in 2017 and 2019 [32,33]. The updated regulations in 2019 included the expanded role of community pharmacists in providing patient care services and outlined several pharmaceutical care services that could be provided in community pharmacies. This is the current active regulation (i.e., the Executive Regulations of the Practice of Health Professions 2019) that provides the expanded scope, as the updated Executive Regulations of the Law of Pharmaceutical Institutions, Pharmaceutical and Herbal Products 2020 did not include these provisions. Another key law is the Law of Narcotics and Psychotropic Substances in Saudi Arabia (2005) and its executive regulations in 2010, which regulated the dispensing of controlled medicines, including narcotic and psychotropic medicines, in the community pharmacy sector [34].

As mentioned, the community pharmacy-regulated scope of practice is currently determined by the Law of Pharmaceutical Institutions, Pharmaceutical, and Herbal Products in Saudi Arabia (2020), its executive regulations (2020) [27], and the Law of the Practice of Health Professions in Saudi Arabia (2005), and its updated executive regulations (2019) [33]. Community pharmacists are now an integral part of the healthcare system and are authorized to provide a wide range of professional activities, services, and products. These include traditional services such as dispensing prescription medicines and supplying OTC medicines, herbal products, and other pharmaceuticals. Moreover, with additional licenses, community pharmacies could provide access to narcotic and controlled medicines according to the Law of Narcotics and Psychotropic Substances in Saudi Arabia (2005) and its executive regulations (2010) [34,35]. Moreover, according to Article 14 of the Law and Executive Regulations of Pharmaceutical Institutions, Pharmaceutical, and Herbal Products (2020), all medicines and pharmaceutical products, including OTC medicines, are exclusively dispensed by pharmacies and are not allowed to be sold outside pharmacies at other outlets. Exceptions could be made for very few OTC medicines to be sold outside pharmacies if deemed necessary upon approval by the Saudi Food and Drug Authority (FDA) [27]. Moreover, registered herbal medicines and products are exclusively dispensed by pharmacies and herbal product licensed shops. However, exceptions could be made for a few herbal products upon approval of the Saudi FDA to be sold at other outlets [27]. Additionally, pharmacists were allowed to perform generic substitutions except for narrow therapeutic index (NTI) drugs according to Article 23 of the Law of the Practice of Health Professions in Saudi Arabia (2005) and its executive regulations [33].

Besides dispensing services, the professional scope includes other professional services such as medication counseling, providing self-care and health-related advice, and guidance and patient education on non-pharmaceutical products [36-39]. Furthermore, community pharmacies are allowed to provide access to a wide range of health-related and personal care products, such as skin care products, oral care products, cosmetics, vitamins, minerals, dietary supplements, and medical equipment and devices [40-49].

In terms of clinical services, community pharmacists had previously limited authority in providing patient care services. However, in 2019, according to Article 13 of the updated executive regulations on the practice of healthcare professionals in Saudi Arabia, community pharmacists were authorized to provide additional patient care services [33]. Consequently, community pharmacists’ scope of practice has expanded to include several patient care services (i.e., advanced services) [33,39,50]. These include patient assessment and monitoring services (vital sign measurements). This service includes measurement of blood pressure, heart rate, temperature, weight, height, body mass index (BMI), waist circumference, and oxygen saturation (SPO2). Another key service includes point-of-care (POC) testing (e.g., blood glucose, glycated hemoglobin (HbA1c), and lipid profile). These services must be delivered in a separate room in the pharmacy to maintain patient privacy. Moreover, medication therapy management (MTM) can be provided in pharmacies. This service includes providing pharmaceutical care for patients with chronic diseases, and it must be delivered in a separate room (i.e., a clinic) in the pharmacy to maintain patient privacy. In addition, modifications in the therapeutic plan must be discussed with the treating physicians for their approval. Moreover, the MTM service should be provided by a registered pharmacist qualified to provide the clinical service [51]. Furthermore, pharmacy-based immunization services are legally allowed in community pharmacies [33,39]. To provide this service, community pharmacists must have additional training and certification in pharmacy-based immunization delivery, and the service must be delivered in a separate room or area in a pharmacy equipped with the requirements for vaccine administration [39]. Vaccines that could be provided in community pharmacies include seasonal influenza (flu), pneumococcal, shingles, hepatitis, human papillomavirus (HPV), tetanus, meningococcal, tetanus, diphtheria, pertussis (Tdap), *Haemophilus influenzae* type b (Hib), and COVID-19 vaccines [39]. 

## Materials and methods

Study design, setting, and population

This cross-sectional descriptive study was conducted with community pharmacists in the Qassim region of Saudi Arabia. The Qassim region was selected for geographical convenience. However, various pharmacies, including major chain pharmacies, exist in this region. Furthermore, the laws and executive regulations governing community pharmacy practices are the same in all administrative regions of Saudi Arabia, as they are national-level laws and regulations [23].

Sample size and sampling procedure

The sampling unit was the community pharmacy, as the study collected data specific to pharmacies (i.e., their products and services). Consequently, in each pharmacy, when there was more than one pharmacist, only one was invited to participate to avoid duplicate responses from the same pharmacy. Convenience sampling was used for this study. The selection of pharmacies was based on the geographical convenience of the data collector. However, to minimize selection bias, several factors were taken into consideration, including the type of pharmacy (i.e., independent, small-chain, and large-chain pharmacies), the location of the pharmacy (i.e., main streets, adjacent to medical polyclinics), and different areas of the region. Based on resources and logistical considerations, 115 pharmacies were visited to collect data. This represented approximately one-quarter of all pharmacies in the Qassim region, as there were 512 pharmacies in 2023 based on the data published by the Ministry of Health [52].

Development of the questionnaire

The questionnaire was developed based on the current scope of practice of pharmacists, as stipulated in laws and regulations [27,33,39]. Additionally, the researcher conducted field visits and discussions with community pharmacists to inform the development of the questionnaire. The questionnaire was thoroughly reviewed to ensure its face and content validity. The initial draft was given to three academics with expertise in community pharmacy practice and quantitative research methods for their comments and feedback. Subsequently, the draft was piloted and tested with five community pharmacists to ensure readability, clarity, and simplicity. The final questionnaire consisted of three sections. The first section comprised the demographic data and practice characteristics of the participants (i.e., sex, age, and qualifications). The second section explored the types of pharmaceuticals and products sold by pharmacies. This section included 15 products, including prescription medicines, OTC medicines, controlled and narcotic medicines, vaccines, herbal products, vitamins, minerals, dietary supplements, medical equipment and devices, etc. The participants were asked to indicate whether each type of product is available in the pharmacy by choosing the option "yes" or "no.". The third section explored the current activities and professional services offered by community pharmacies. This section included 13 services and activities, including medication counseling, e-prescribing service, provision of health advice, management of minor ailments, vaccination services, POC testing, smoking cessation, etc. The participants were asked to indicate whether each service is offered in the pharmacy by choosing the option "yes" or "no.". As mentioned earlier, the selection of these products and services was based on the existing laws and regulations determining the scope of practice, field visits, and consultations with community pharmacists. 

Data collection procedures

A web-based survey was created using an online survey platform (SurveyMonkey, San Mateo, CA). Subsequently, the researcher prepared an invitation letter along with an electronic link and barcode to access the online survey. The invitation letter included brief information about the study, instructions on how to fill in the questionnaire, and how to submit the online questionnaire. Participants were informed that their participation was entirely voluntary and assured of the anonymity of their responses. After an appropriate orientation, a data collector visited the community pharmacies to invite them to participate in the study and provided them with an invitation letter.

Data management and analysis

The data collected were downloaded from Microsoft Excel sheets (Microsoft Corp., Redmond, WA) from the platform. The data were checked and coded. The analysis included descriptive statistics, namely frequencies and percentages.

Ethical approval

This study was conducted in accordance with the ethical principles of the Declaration of Helsinki. Ethical approval was granted by the Regional Research Ethics Committee of the Qassim Region of Saudi Arabia (approval no. 607/46/210).

## Results

Response rate

The data collector visited 115 community pharmacies. Of these, one declined to participate in the study, five received the survey but did not submit responses, and 109 received the survey and submitted their responses. The response rate was 94.78%. 

Participants’ demographic data and characteristics

Most participants (n = 106; 97.25%) were men. The participants were from varying age groups, with 46 (42.20%) in the age group of 31-40 years, followed by the age group of 23-30 years old (n = 38; 34.86%) and the age group of 41-50 years (n = 22; 20.18%). Most participants held a bachelor’s degree in pharmacy (n = 71; 65.14%). Most pharmacies were run by ≤ two pharmacists (n = 76, 69.72%). The results are summarized in Table 1. 

**Table 1:** Participants’ demographic data and characteristics

Variable	Number (%)
Sex	
Male	106 (97.25)
Female	3 (2.75)
Age (in years)	
23–30	38 (34.86)
31–40	46 (42.20)
41–50	22 (20.18)
> 50	3 (2.75)
Highest degree or qualification	
Bachelor's degree	71 (65.14)
Doctor of Pharmacy	22 (20.18)
Postgraduate degree	16 (14.68)
Number of pharmacists working in the pharmacy	
≤ 2	76 (69.72)
3–4	30 (27.52)
≥5	3 (2.75)

Types and availability of pharmaceuticals, medicines, vaccines, and products in pharmacies 

In this study, all pharmacies (n = 109, 100%) provided prescription medicines, OTC medicines, vitamins, minerals, and dietary supplements. However, only a few pharmacies had controlled narcotic medicines (n = 6; 5.50%) and vaccines (n = 4; 3.67%). The vast majority of pharmacies provided access to and supplied patients and customers with herbal products (n = 106; 97.25%), self-diagnostic test/home-test kits (n = 106; 97.25%), first-aid kits (n = 104; 95.41%), and medical equipment and devices and their accessories (n = 98; 89.91%). All pharmacies (n = 109; 100%) sold health-related products and essentials, including oral care products, skincare products, hair care products, cosmetics, baby care products, baby milk, food, and other hygiene and personal care products. Table 2 presents the results of the study. 

**Table 2:** Types and availability of pharmaceuticals, medicines, vaccines, and products in pharmacies

S.no.	Product	Availability number (%)	
		Yes	No
1	Prescription medicines	109 (100.00)	0 (0.00)
2	Over-the-counter (OTC) medicines	109 (100.00)	0 (0.00)
3	Vitamins, minerals, and dietary supplements	109 (100.00)	0 (0.00)
4	Controlled and narcotic medicines	6 (5.50)	103 (94.50)
5	Vaccines	4 (3.67)	105 (96.33)
6	Herbal products	106 (97.25)	3 (2.75)
7	Medical equipment and devices (e.g., for blood pressure, glucose measurement device, and their accessories)	98 (89.91)	11 (10.09)
8	Self-diagnostic test/At home-test kits (e.g., pregnancy test or others)	106 (97.25)	3 (2.75)
9	First aid kits	104 (95.41)	5 (4.59)
10	Oral care products (e.g., toothpaste, mouthwash)	109 (100.00)	0 (0.00)
11	Skincare products	109 (100.00)	0 (0.00)
12	Hair care products	109 (100.00)	0 (0.00)
13	Beauty products and cosmetics	109 (100.00)	0 (0.00)
14	Baby care products, baby milk and food, and related accessories	109 (100.00)	0 (0.00)
15	Hygiene and personal care products	109 (100.00)	0 (0.00)

Types and availability of pharmaceutical services and activities in pharmacies

In this study, all pharmacists (n = 109; 100%) indicated that they had provided medication counseling. However, the e-prescription service (Wasfaty) was provided in only 55.96% (n = 61) of pharmacies. Most pharmacists stated that they provided health education and promotion (n = 104; 95.41%), management of minor ailments (n = 97; 88.99%), and patient training on the use of medical devices (n = 101; 92.66%). Other pharmaceutical activities included travel health advice and consultations (n = 57; 52.29%) and smoking cessation (n = 34; 31.19%). However, patient care services, including vaccination services (n = 1; 0.92%), patient assessment and monitoring services (n = 1; 0.92%), and POC testing (n = 1; 0.92%), were limited. 

Other services provided by community pharmacies included online shopping through the online store of the pharmacy, pharmacy app, or hotline (n = 73; 66.97%), and home delivery of medicines (n = 59; 54.13%). In this study, 70.64% (n = 77) of the pharmacies provided instant/live chat communication (e.g., WhatsApp or other methods). Table 3 presents the results. 

**Table 3:** Types of pharmaceutical activities and services in the pharmacies

S.no.	Service	Availability number (%)	
		Yes	No
1	Medication counseling	109 (100.00)	0 (0.00)
2	E-prescribing (Wasfaty) service	61 (55.96)	48 (44.04)
3	Provision of health advice (health education and promotion)	104 (95.41)	5 (4.59)
4	Management of minor ailments	97 (88.99)	12 (11.01)
5	Vaccination service	1 (0.92)	108 (99.08)
6	Patient assessment and monitoring service/pharmacist-led service (i.e., vital signs measurement service: Blood pressure, temperature, weight, waist circumference, heart rate, oxygen saturation (SPO2), etc)	1 (0.92)	108 (99.08)
7	Point-of-care (POC) testing	1 (0.92)	108 (99.08)
8	Patient training on the use of medical devices (e.g., use of glucometer, blood pressure device/monitor, nebulizers)	101 (92.66)	8 (7.34)
9	Smoking cessation	34 (31.19)	75 (68.81)
10	Travel health advice/consultation	57 (52.29)	52 (47.71)
11	Home delivery of medicines	59 (54.13)	50 (45.87)
12	Online shopping (through the online store of the pharmacy or pharmacy app or hotline)	73 (66.97)	36 (33.03)
13	Instant/live chat communication with the pharmacy (e.g. WhatsApp or other methods)	77 (70.64)	32 (29.36)

## Discussion

The study findings showed that community pharmacies play an essential role in Saudi Arabia’s healthcare system. They provide many essential pharmacy services, including dispensing prescription medicines, medicine counseling, access to OTC medicines and self-care therapeutics, health advice and promotion, herbal products, vitamins, and dietary supplements, self-diagnostic tests/kits, medical equipment and devices/monitors, and relevant training to use these devices/monitors to meet the patient’s needs and requirements. Additionally, they provide convenient access to cosmetics, cosmeceuticals, products related to personal care, oral care, and other daily essentials for clients and patients to promote their health and wellness. Moreover, other services and contributions to public health by community pharmacists in Saudi Arabia have expanded in recent years, including smoking cessation [53,54]. Similar to studies from other countries [55-57], community pharmacists are increasingly involved in providing travel health advice to their patients and customers. Consequently, patients and clients in Saudi Arabia visit community pharmacies for a variety of services and products, including dispensing prescription medicines, prescription refills, supply of OTC medicines, management of minor health conditions (i.e., self-care therapeutics), and seeking health advice and information on medicines, medical devices, first aid information, vitamins and nutritional products, cosmetics, baby care products, and other health-related products [36,58-60]. This made community pharmacies vital places visited frequently by communities for pharmaceutical and non-pharmaceutical products and provided multiple opportunities for interactions with pharmacists. Moreover, it has diversified the revenue of pharmacies beyond medicine to include other widely used health and wellness products. This helps generate profit and financial sustainability in the sector [61]. According to laws and regulations in Saudi Arabia, prescription medicines, controlled medicines, and OTC medicines are exclusively dispensed via pharmacies. Very few exceptions can be made to OTC products upon approval by the Saudi FDA. Herbal products are sold exclusively via pharmacies and herbal product-licensed shops, except for those approved by the Saudi FDA [27]. Furthermore, pharmacists are the only healthcare professionals who are allowed to dispense prescriptions [33]. Consequently, community pharmacists contribute to access to medicines and healthcare in the community setting and are widely accessible and easily approached by the community for their services and expertise in medication therapy.

Recent additional services provided by community pharmacies include home delivery of medicines and products, shopping via online stores of pharmacies and mobile apps, online consultations, and communication services (e.g., live chat). These services have been increasingly promoted and have witnessed huge growth since the COVID-19 pandemic, which has accelerated the adoption of telehealth and digital solutions in community pharmacies in Saudi Arabia [62-64]. According to a recent survey of community pharmacists in Saudi Arabia, 71.4% of the study participants believed that medicine delivery services help patients continue taking their medications and improve medication adherence, 91% believed that community pharmacists could provide virtual consultations via online platforms, and 81.4% believed that home medicine delivery applications would become a common practice in the future and would gradually replace the traditional method of in-person medicine collection from pharmacies [64]. Globally, many community pharmacists have adopted telepharmacy and home delivery services to ensure the provision of services and continuity of care to their patients, especially during the lockdown periods and for COVID-19-suspected patients or high-risk individuals [64].

One of the key initiatives introduced in recent years in Saudi Arabia is the nationwide electronic prescribing service through community pharmacies (my prescription Wasfaty service). This initiative expanded the scope of the community pharmacy sector in Saudi Arabia. Community pharmacists are now integrated into primary care services by dispensing prescriptions and providing services to patients from primary care centers and outpatients in many hospitals [65]. Community pharmacies have replaced pharmacies in government primary care centers in Saudi Arabia. In this study, 55.96% of the surveyed community pharmacies indicated that they were authorized providers of Wasfaty services. This rate is similar to the national rate at which pharmacies provide these services. As of July 2024, 5053 community pharmacies are authorized providers of Wasfaty services [22], representing approximately 50% of all community pharmacies in 2023 (10189 pharmacies) [66]. However, the service has expanded, with more pharmacies joining it since its introduction in 2018/2019. Several recent studies have assessed community pharmacists' and patients’ perspectives on this nationwide service [65, 67, 68]. From the pharmacists’ perspective, the service helps improve prescribing practices and could further improve medication safety and reduce medication dispensing errors [69]. From the patients’ perspective, they were satisfied with the service in terms of accessibility to community pharmacies, pharmacy facilities, pharmacists’ knowledge and skills, and the instructions and counseling provided [67]. Other studies reported similar findings and satisfaction with the service, including time flexibility in dispensing e-prescriptions, easier and more convenient access to pharmacies, waiting time, medication counseling, and prescriptions [65, 70]. In this study, only a few pharmacies sold the vaccines. This is explained by the fact that vaccination services are still very limited in pharmacies across the country [71]. Additionally, only 5.50% of pharmacies provided access to controlled medicines. This is similar to the national percentage, as approximately 360 pharmacies nationwide are listed in the Saudi FDA directory as pharmacies providing these medicines [35]. This represents approximately 3.53% of the total number of pharmacies by 2023. This is because access to these medicines is typically provided by government health services. In addition, this service requires separate licenses, additional security requirements, and detailed procedures. 

The practice of community pharmacies in Saudi Arabia is still focused on traditional roles (i.e., dispensing, supplying medicines and products, medication counseling, guidance, and patient education on non-pharmaceutical products) [36-39]. Moreover, the study findings showed that clinical services are still limited. This is similar to other findings in the literature showing that direct patient care services, such as immunization services, POC testing, MTM, screening, and disease management, are limited to the vast majority of community pharmacies in Saudi Arabia [20,72-74]. This is because of several factors and barriers to the provision of clinical services. These include lack of access to patient data (patients' records) [72], lack of pharmacy support staff (i.e., pharmacy technician) [72,75,76], lack of public awareness of the roles of community pharmacists and their potential in providing clinical service [60,72,77], lack of time and heavy workload due to shortage of pharmacists [53,75,76,78,79], lack of designated area for providing the service/privacy [53,60,75,78,80], lack of adequate clinical training or continuing professional developments to provide the services (knowledge and skills) [53,76,78,80,81], and limited coordination and communication with other healthcare providers (i.e., lack a model of practice in primary care or collaborative practice agreements) [75,80,82]. Consequently, it is vital to overcome these barriers to ensure that pharmacists provide more clinical services to patients.

This study has several implications for practice, policy, and future research. This study showed that community pharmacists play crucial roles in primary care and public health, including a recent expansion in their role in dispensing and refilling prescriptions and providing pharmaceutical services via nationwide e-prescription services for patients from public health centers and outpatients from hospitals. However, beyond dispensing services and standard patient counseling, patient care services are still limited in pharmacies, despite recent regulations that allow such services. Consequently, health policymakers and pharmacy leaders should address the challenges and barriers hindering the implementation of these services. This is particularly important, building on the recent trends in healthcare transformation policies in Saudi Arabia that put more emphasis on preventive healthcare services and the involvement of the private sector in the delivery of healthcare. A comprehensive strategy involving all stakeholders is required to facilitate patient-care services in pharmacies. This includes addressing the current barriers reported widely in the literature, such as lack of access to patient data, lack of support staff (i.e., pharmacy technicians and assistants), and pharmacy capacity to provide services (i.e., the number of pharmacists per pharmacy), and establishing a model of practice to integrate the service into the primary care system. Additionally, collaborative practice agreements (CPAs) in community pharmacy settings can help expand clinical services. This will help utilize the experience and skills of community pharmacists in providing patient care and reduce the burden on primary care services at other institutions. This is because more pharmacists with a six-year PharmD degree are joining this sector [24]. Future research could examine the quality of pharmacy services and explore the facilitators and barriers to providing patient care services from the perspective of all stakeholders. Moreover, patient willingness to utilize these services should be investigated based on recent developments in the pharmacy profession.

Strengths and limitations

This study has several strengths. To my knowledge, this is the first study to comprehensively examine the types and availability of pharmaceuticals, products, professional services, and activities in community pharmacies in Saudi Arabia. This study had a high response rate (94.78%). This is because the study utilized in-person visits to community pharmacies by a trained data collector to invite pharmacists and provide them with a brief overview. However, this study has some limitations. Convenience sampling was used in this study. Although inevitable owing to logistical and resource constraints, this could affect the generalization of the results to the entire region or country. To address this and enhance the representativeness of the study, various community pharmacies were visited, including independent, small-chain, and large-chain pharmacies. In addition, as this is a cross-sectional study, the findings reflect the current state of pharmacies. Consequently, future studies should be conducted to monitor the progress and development of the services in the community pharmacy sector. Given the limited literature on the current pharmaceutical activities of community pharmacies in Saudi Arabia, the study findings are valuable and could be used to provide future guidance to health decision-makers and pharmacy leaders to improve community pharmacy practices and services, building on the recent developments of the profession.

## Conclusions

The study findings show that community pharmacies in Saudi Arabia play a crucial role in the healthcare system. These include dispensing and refilling prescriptions and providing access to OTC medicines and self-care therapeutics, herbal medicines, vitamins, minerals, dietary supplements, medical equipment and devices, and a variety of pharmaceuticals and products related to health and wellness. Community pharmacies provide several services, including medication counseling, e-prescribing (Wasfaty) service, management of minor ailments, health advice, patient education, and other public health activities. In addition, other growing services included online shopping and home delivery of medicines and products. However, their clinical services are limited. Consequently, a holistic strategy involving all stakeholders is required to further enhance the role of community pharmacists and better utilize their skills and training in providing preventive healthcare services and optimizing medication therapy in primary care settings.
